# ATTRIBUTED DIGNITY ACROSS THE LIFESPAN: COMPARING DIGNITY IN OLDER ADULTS AND COLLEGE-AGE INDIVIDUALS

**DOI:** 10.1093/geroni/igae098.4124

**Published:** 2024-12-31

**Authors:** Emefa Adawudu, Chunfang Chen, Jeungok Choi, Cynthia Jacelon

**Affiliations:** University of Massachusetts, Amherst, Amherst, Massachusetts, United States; University of Mount Saint Vincent, USA, University of Mount Saint Vincent, USA, New York, United States; University of Massachusetts, Amherst, Amherst, Massachusetts, United States; University of Massachusetts, Amherst, Amherst, Massachusetts, United States

## Abstract

Attributed Dignity is a core value in human interaction. Researchers have shown that the perception of dignity changes across the lifespan. To understand attributed dignity across the lifespan, we compared the factor structure of the Jacelon Attributed Dignity Scale (JADS) between samples of older adults and college-aged individuals. The theory of attributed dignity guided this study. Data was collected from 289 older adults from senior centers in New England and 380 college students at a large public university in New England using JADS, social desirability scale (SDS), and Rosenberg Self Esteem Scale (SES). SDS and SES were used to determine construct validity. Exploratory Factor Analysis (EFA), construct validity, and reliability of the scale were analyzed. EFA resulted in an 18-item scale with four factors including ‘self-value’, ‘perceived value from others’, ‘behavior with respect towards others’, and ‘self in relation to others’ for both older adults and college-age individuals. Older adults rated ‘behavior with respect towards others’ as the most important factor, while ‘self-value’ was considered the most important attribute of dignity in college-age individuals. Correlations between the JADS, SDS, and SES indicate attributed dignity is a unique concept. Findings indicate that the perception of attributed dignity varies across the lifespan. Consistent with a developmental perspective, older adults value their behavior most highly, while college-aged individuals are more focused on their own self-value. Understanding how the emphasis among factors of attributed dignity changes across the lifespan can provide direction for interventions to effectively enhance attributed dignity.

